# Public health and economic impact of switching from a trivalent to a quadrivalent inactivated influenza vaccine in Mexico

**DOI:** 10.1080/21645515.2019.1678997

**Published:** 2019-12-18

**Authors:** Guillermo M. Ruiz-Palacios, John H. Beigel, Maria Lourdes Guerrero, Lucile Bellier, Ramiro Tamayo, Patricia Cervantes, Fabián P. Alvarez, Arturo Galindo-Fraga, Felipe Aguilar-Ituarte, Juan Guillermo Lopez

**Affiliations:** a Department of Infectious Diseases, Instituto Nacional de Ciencias Médicas y Nutrición Salvador Zubirán, Mexico City, Mexico; b Division of Microbiology and Infectious Diseases, National Institute of Allergy and Infectious Diseases (NIAID), National Institutes of Health, Bethesda, MD, US; c Creativ-Ceutical, London, UK; d Sanofi LATAM, Mexico City, Mexico; e Sanofi Pasteur, Lyon, France; f Subdirección de Epidemiología Hospitalaria y Control de Calidad de la Atención, Instituto Nacional de Ciencias Médicas y Nutrición Salvador Zubirán, Mexico City, Mexico

**Keywords:** Budget impact, cost, economic analysis, influenza, influenza B virus, Mexico, public health impact, seasonal influenza, vaccination, vaccine

## Abstract

Most influenza vaccines in Mexico are trivalent, containing two influenza A strains and a single B strain. Quadrivalent influenza vaccines (QIVs) extend protection by including an additional B strain to cover both co-circulating B lineages. Here, we retrospectively estimated how a switch to QIV in Mexico would have impacted influenza-related health outcomes over the 2010/2011 to 2015/2016 influenza seasons, and prospectively estimated the budget impact of using QIV in Mexico’s national immunization program from 2016/2017 to 2020/2021. For the retrospective estimation, we used an age-stratified static model incorporating Mexico-specific input parameters. For the prospective estimation, we used a budget impact model based on retrospective attack rates considering predicted future vaccination coverage. Between 2010/2011 and 2015/2016, a switch to QIV would have prevented 270,596 additional influenza cases, 102,000 general practitioner consultations, 140,062 days of absenteeism, 3,323 hospitalizations, and 312 deaths, saving Mex$214 million (US$10.8 million) in third-party payer costs. In the prospective analysis, a switch to QIV was estimated to prevent an additional 225,497 influenza cases, 85,000 general practitioner consultations, 116,718 days of absenteeism, 2,769 hospitalizations, and 260 deaths, saving Mex$178 million (US$9 million) in third-party payer costs over 5 years. Compared to the trivalent vaccine, the benefit and costs saved with QIV were sensitive to the distribution of influenza A vs. B cases and trivalent vaccine effectiveness against the mismatched B strain. These results suggest switching to QIV in Mexico would benefit healthcare providers and society by preventing influenza cases, morbidity, and deaths, and reducing associated use of medical resources.

## Introduction

Influenza epidemics are caused by influenza A and B viruses, which co-circulate worldwide.^1^ In Mexico, influenza epidemics occur almost every winter and result in substantial mortality and a considerable burden to healthcare resources.^2,3^ To protect those most vulnerable to influenza and its complications, the Mexican health authority recommends vaccination ahead of each influenza season for children aged 6 months to 4 years, adults aged ≥ 60 years, and individuals with chronic conditions.^4^

Most influenza vaccines in Mexico are trivalent, containing two strains of influenza A (H1N1 and H3N2) and a single B strain.^5^ However, since the early 1980s, two genetically distinct lineages of influenza B virus, Yamagata and Victoria, have co-circulated globally.^6^ This has complicated the selection of the correct B lineage to include in the trivalent influenza vaccine (TIV) ahead of each season, and has resulted in frequent mismatches between the B lineage in the vaccine and the predominant B lineage in circulation.^6–8^ Quadrivalent influenza vaccines (QIVs), that include one strain from each B lineage, have been developed to help overcome this problem with TIV and to reduce influenza B cases in seasons where both lineages co-circulate or where the predominant B strain is of the alternate lineage to that selected for the TIV.^6^

The health economic impact of a switch from TIV to QIV has been evaluated for several countries worldwide using retrospective and prospective economic models. In a systematic review of 16 health economic evaluations from high-income countries, replacing TIV with QIV provided considerable health benefits through reducing influenza-related morbidity and mortality within a range of 0.15%−6.5%.^9^ In addition, a switch to QIV was estimated to save costs to healthcare systems and society that compensated for a higher unit price of QIV. However, because almost all studies have focused on high-income settings, the public health and economic benefits of QIV in low- and middle-income countries are less clear.^10^ In one analysis, QIV provided a greater reduction in influenza-related morbidity than TIV in communities in South Africa (middle-income) and Vietnam (low-income) as compared with Australia (high-income). However, QIV was only cost-effective in these low- and middle-income communities when a high influenza attack rate was assumed, and its superiority over TIV depended on the influenza B burden, the rates of vaccine mismatch, the levels of comorbidities in the population, and unit costs.^11^

The health and economic impacts of replacing TIV with QIV have not been previously evaluated in Mexico. The objectives of this study were (a) to retrospectively estimate the impact that QIV would have had on influenza-related health outcomes over six previous influenza seasons if it had been used instead of TIV in Mexico; and (b) to prospectively estimate the budget impact of QIV in Mexico over five future seasons after its introduction into the national immunization program.

## Methods

### Models

**Figure 1.**
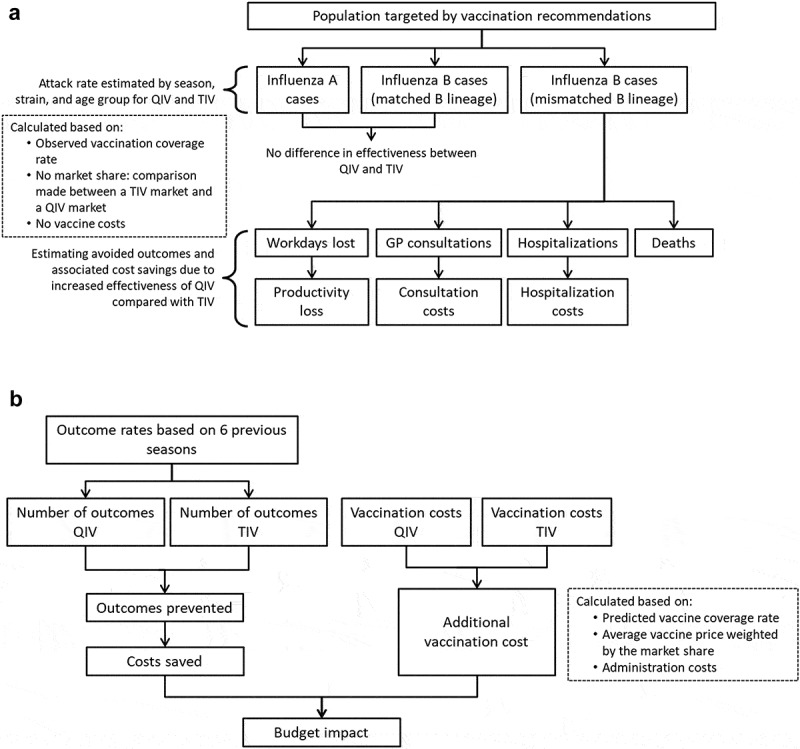
Model structure. (A) Retrospective public health model. (B) Prospective budget impact model. Abbreviations: GP, general practitioner; QIV, quadrivalent influenza vaccine; TIV, trivalent influenza vaccine.

An age-stratified static public health impact model was developed using Excel VBA (Microsoft, Redmond, WA, US) to retrospectively estimate the impact a switch from TIV to QIV would have had for the influenza seasons 2010/2011 to 2015/2016 (Figure 1A). We chose this time horizon to adequately reflect the wide variability in the circulation and burden of influenza between seasons. The model was based on the methodology presented by Reed et al.^12^ and incorporated Mexico-specific inputs wherever possible. A separate incidence-based, predictive model was developed to prospectively estimate the budget impact of a switch to QIV in Mexico for the seasons 2016/2017 to 2020/2021, by incorporating the attack rates calculated in the retrospective analysis model and considering predicted future vaccination coverage (Figure 1B).

To match the population recommended for influenza vaccination in Mexico,^4^ the model population was stratified into the following age groups: ≤ 4 years, 5−17 years, 18−49 years, 50−59 years, and ≥ 60 years, and only individuals with comorbidities considered risk factors for influenza complications were included in the population aged 5–59 years.

#### Retrospective public health impact model

The retrospective model calculated the epidemiological and economic burden that could have been avoided had QIV been used instead of TIV for the influenza seasons 2010/2011 to 2015/2016. To do so, two scenarios were analyzed: (a) a real-life scenario using data on the observed burden of influenza in these seasons with TIV vaccination; (b) a hypothetical scenario that simulated the number of influenza-outcomes that would have occurred if the population had been vaccinated with QIV instead of TIV. Epidemiological outcomes included influenza cases and influenza-related GP consultations, hospitalizations, and deaths. Economic outcomes included costs associated with influenza-related GP consultations and hospitalizations, and productivity losses. For each season and each outcome, the model calculated the outcome rate attributable to each strain in an unvaccinated population, and then applied the average vaccination coverage and strain-specific vaccine efficacy to generate strain-specific outcome rates in the population vaccinated with TIV and QIV. Overall outcome rates were then calculated for each vaccine by adding the three individual strain-specific rates, and applied to the population size to obtain the total number of outcomes in the TIV and QIV scenarios. For each season, the burden of influenza that could have been avoided was calculated by subtracting the outcomes of the TIV scenario from those of the QIV scenario to give the influenza cases (and associated resource use and costs) caused by the B strain not included in TIV. Economic assessments explored costs from the third-party payer and societal perspectives. From the third-party payer perspective, costs were included from GP visits and hospitalizations. The societal perspective included these costs as well as the cost of workdays lost to influenza by the individual or their caregiver. Differences between the costs generated in TIV and QIV scenarios were calculated for each cost category. The total cost saved with QIV was also calculated.

#### Prospective budget impact model

The prospective budget impact model calculated the predicted influenza incidence and associated outcomes of a switch to QIV in Mexico for the influenza seasons 2016/2017 to 2020/2021. Influenza-related outcome rates were based on those observed from the previous seasons in the retrospective model. The age- and risk-specific outcome rates observed during each past season were adjusted to obtain the rates for a fully unvaccinated population, a population fully vaccinated with TIV, and a population fully vaccinated with QIV. These rates were then projected over each prospective season assuming constant vaccination coverage. The average numbers of outcomes per projected year were then calculated for each vaccination scenario (unvaccinated, vaccinated with TIV, or vaccinated with QIV) and cumulated over 5 years.

### Model inputs

Model inputs and assumptions are presented in Table 1 and Supplemental Table 1. Data from Mexico were preferred, but when unavailable, data from other countries were used.

**Table 1.** Input values.

	Input		DSA range	
	Value	Reference	Range	Reference
Population, n				
≤4 years^a^	9,931,368	^13^	−	−
5−17 years (high-risk)	5,813,464	^13^	±20%	−
18−49 years (high-risk)	24,149,671	^13^	±20%	−
50−59 years (high-risk)	6,378,002	^13^	±20%	−
≥60 years	12,973,411	^13^	−	−
Observed vaccination coverage, %				
≤4 years	67.9^b^	^14^	42.7−78.5^c^	^14^
5−17 years	56.0	^14^	42.7−78.5^c^	^14^
18−49 years	56.0	^14^	42.7−78.5^c^	^14^
50−59 years	56.0	^14^	42.7−78.5^c^	^14^
≥60 years	62.4	^15^	49.1−78.5^c^	^15^
Predicted vaccination coverage, %				
≤4 years	67.9	^14^	42.7−78.5^c^	^14^
5−17 years	56.0	^14^	42.7−78.5^c^	^14^
18−49 years	56.0	^14^	42.7−78.5^c^	^14^
50− 9 years	56.0	^14^	42.7−78.5^c^	^14^
≥60 years	62.4	^15^	49.1−78.5^c^	^15^
Vaccine efficacy against influenza A, %^d^				
≤4 years	59.0	^16^	−	−
5−17 years	61.0	^16^	−	−
18−49 years	61.0	^16^	−	−
50−59 years	61.0	^16^	−	−
≥60 years	58.4	^16^	−	−
Vaccine efficacy against matched B, %^d^				
≤4 years	66.0	^16^	−	−
5−17 years	77.0	^16^	−	−
18−49 years	77.0	^16^	−	−
50−59 years	73.0	^16^	−	−
≥60 years	69.5	^16^	−	−
Vaccine efficacy against mismatched B, %^d,e^				
≤4 years	44.0	^16^	35.0−53.0	^16^
5−17 years	52.0	^16^	42.0−62.0	^16^
18−49 years	52.0	^16^	18.0−94.0	^16^
50−59 years	49.0	^16^	18.0−96.0	^16^
≥60 years	47.2	^16^	16.0−99.0	^16^
Vaccine cost, Mex$				
TIV	62.00	^17^	±20%	−
QIV^f^	62.00	−	±20%	−
Cost of vaccine administration, Mex$	19.01	^18^	14.64−23.57^g^	^18^
Cost of GP visit, Mex$	674.00	^19^	±20%	−
Cost of hospitalization, Mex$				
≤4 years	31,200.80	^19,20^	26,847.20−35,554.40	^19,20^
5−17 years	42,084.00	^19,20^	34,828.80−54,420.00	^19,20^
18−49 years	42,084.00	^19,20^	34,828.80−54,420.00	^19,20^
50−59 years	54,420.00	^19,20^	47,889.60−72,560.00	^19,20^
≥60 years	58,773.60	^19,20^	53,268.03−75,215.14	^19,20^
Workdays lost^h^, N				
≤4 years^i^	0.84	^21^	0.50−1.25	−
5−17 years^i^	0.84	^21^	0.50−1.25	−
18−49 years	2.70	^21^	2.00−4.00	−
50−59 years	2.42	^21^	2.00−4.00	−
≥60 years	1.27	^21^	1.00−2.00	−
Daily wages, Mex$	333.23	^22^	−	−

The population aged 5–59 years represents high-risk individuals only. Abbreviations: DSA, deterministic sensitivity analysis; GP, general practitioner; QIV, quadrivalent influenza vaccine; TIV, trivalent influenza vaccine.

^a ^The number of persons aged 6–12 months was estimated by dividing by two the size of the population aged 0 years.

^b ^The rate for the ≤ 4 years group was calculated as the average of the rate for children aged 6–11 months and the rate for children aged 12–35 months. This average was then weighed by the population size in each age group used in the original data.^14^

^c ^For 0−59-year-olds, the lower bound of the 95% confidence interval for the coverage rate of 6–11-month-olds from Gutierrez et al. was used.^14^ For ≥ 65-year-olds, the weighted average of the lower bounds of the 95% confidence interval for 60–64- and ≥65-year-olds from Cruz-Hervert et al. was used.^15^ For all age groups, the upper bound used was that from the reported coverage rate of ≥65-year-olds by the Organization for Economic Co-operation and Development.^23^

^d ^Vaccine efficacy represents the % reduction in the incidence of laboratory-confirmed influenza among vaccinated individuals compared to unvaccinated individuals in the same randomized clinical trials.

^e ^For TIV only.

^f ^The price of QIV was assumed to be the same as the price of TIV.

^g ^For the lower bound, the cost of rotavirus vaccine administration was used from De la Hoz-Restrepo et al.^18^ For the upper bound, the cost of pneumococcal vaccine administration was used from the same study.

^h ^The number of days lost to influenza was adjusted by age-specific economic activity rates from the 2010 Census.^24^

^i ^Workdays lost by children reflect those lost by their caregivers.

#### Model population

Population counts were derived from the Consejo Nacional de Población (CONAPO) database^13^ for the year 2017. The selection of comorbidities among the population aged 5−59 years was based on the 2015 influenza vaccination recommendation of the Mexican Secretariat of Health (Supplemental Table 2).^4^ Where possible, the size of the population with each comorbidity was calculated from national incidence or prevalence data and corrected for persons with more than one comorbidity. To obtain the probability of a condition in a given age group, the size of the population affected by a condition in the age group was divided by the total size of the age group. Correlations between different conditions were not taken into account and were assumed to be independent.

#### Influenza virus circulation

The proportion of influenza A, B/Victoria, and B/Yamagata from reported influenza cases, and the B-strain included in TIV in each season, were used to estimate the number of cases and other influenza-related outcomes in both models (Supplemental Table 1). The distribution of cases by strain and lineage was calculated based on two assumptions: (a) cases not caused by an A strain were assumed to be caused by a B strain, and (b) cases caused by a B strain not of the Yamagata lineage were assumed to be caused by a B/Victoria lineage strain, and vice versa. The proportions of cases caused by A strains (H1N1 and H3N2) during the seasons 2010/2011 to 2015/2016 were obtained from the FluNet database^25^ and data from the Mexico Emerging Infectious Diseases Clinical Research Network (LaRed). For the proportions of cases caused by the B/Victoria and B/Yamagata lineages, unpublished data from the LaRed network were used (based on RT-PCR-tested nasopharyngeal samples collected from influenza-like illness in six Mexican hospitals) for the seasons 2010/2011, 2012/2013, 2013/2014, and 2015/2016. Because LaRed data were not available for Mexico, data from the US^26,27^ were used for the seasons 2011/2012 and 2014/2015.

#### Outcome rates

Influenza attack rates and outcome rates per age group and season are given in Supplemental Table 1. The average influenza attack rates for each age group were derived from the placebo arms of previous influenza vaccine trials.^28–30^ To obtain the rates for each season, the average attack rates were adjusted by a season severity coefficient, equivalent to the ratio of the number of influenza cases reported in a given season over the total number of influenza cases reported during the modeled period (source data from the FluNet database^25^). The influenza-related general practitioner (GP) consultation rate, hospitalization rate, and death rate were derived from estimates from the US population^31^ because data for Mexico were not publically available. GP consultation rate estimates were adjusted by the ratio between the number of consultations per capita in Mexico and the US, obtained from 2011 statistics.^23^ Hospitalization and death rate estimates were not corrected for Mexico as it was assumed these are comparable to the US. Because specific data were not available, influenza-related hospitalization and death rate estimates from the general population in Mexico were conservatively applied to high-risk individuals aged 5–59 years. Finally, each rate estimate was multiplied by the seasonal influenza attack rates to generate rates for each season.

#### Vaccination coverage rates

Vaccination coverage rates for each age group were derived from national surveillance data^15,32^ and were assumed to be constant across seasons and the same for TIV and QIV. Since no specific estimates were available for high-risk individuals aged 5–59 years, the coverage in the 60–64 year group was applied.

#### Vaccine efficacy

Vaccine efficacy against influenza A and B strains was derived from Clements et al.^16^ QIV was assumed to have equal efficacy to TIV against influenza A strains and the shared B strain. TIV efficacy against influenza B in each season depended on whether the B lineage in the vaccine was matched or mismatched with the predominant circulating B lineage. The B-matched vaccine efficacy was used for QIV for all seasons. To comply with the age grouping used in the model, the efficacy estimates from Clements et al.^16^ were adjusted by the number of years in each age group, assuming the population within each age group was uniformly distributed.

#### Resource use and costs

The price of QIV was assumed to be the same as the price of TIV. Vaccine administration costs were based on cost estimates by De La Hoz-Restrepo et al.^18^ The average daily wage used to calculate productivity losses was equal to the average daily salary in Mexico in 2017, obtained from the Secretariat of Labor and Social Welfare.^22^ Workdays lost to influenza were adjusted by age-specific economic activity rates from the 2010 Census.^24^ Where required, costs were converted between Mex$ and US$ using the average exchange rate for 2017 (1 US$ = 19.679 Mex$).^33^ No inflation was applied.

### Deterministic sensitivity analyses

For both models, the sensitivity of the results was explored by varying key parameters within plausible ranges (given in Table 1). The proportion of high-risk individuals and circulating influenza strains, influenza attack rates, and influenza-related GP consultations, hospitalizations, and deaths were each varied by ± 20%. The lower and upper bounds for other parameters were chosen based on confidence intervals, values found from other data sources, or assumptions (see Table 1). The results of the sensitivity analyses are presented as Tornado charts. All analyses were performed using Excel VBA (Microsoft, Redmond, WA, US).

## Results

### Impact of QIV in previous influenza seasons

In the retrospective model, the base case analysis showed that over the six influenza seasons from 2010/2011 to 2015/2016, replacing TIV with QIV would have prevented 270,596 additional influenza cases, 102,000 general practitioner (GP) consultations, 140,062 days of absenteeism, 3,323 hospitalizations, and 312 deaths (Table 1). Young children aged 0 to 4 years would have benefited the most from a switch to QIV, this age group accounting for 43% of the avoided influenza cases, 36% of avoided influenza-related GP consultations, and 49% of avoided hospitalizations (Supplemental Table 3). Adults aged ≥ 60 years also benefited substantially, since 91% of the prevented deaths were within this age group.

The events avoided with QIV resulted in considerable cost savings. A switch from TIV to QIV saved an estimated Mex$213,510,146 (US$10,849,644) from the third-party payer perspective and Mex$260,182,996 (US$13,221,353) from the societal perspective (Table 2). Most of the cost savings were related to avoided influenza-related hospitalizations (68% of total third-party payer costs saved, 56% of total societal costs saved). Of all the cost savings under the third-party payer perspective, 36% were saved in children ≤4 years of age and 37% in adults aged ≥ 60 years (Supplemental Table 3). These proportions were slightly lower under the societal perspective (33% for children aged ≤ 4 years, 33% in adults aged ≥ 60 years) because the productivity losses were included and the workdays lost by parents of a sick child and by older adults were lower than for adults.

**Table 2.** Retrospective outcomes prevented and costs saved by switching from TIV to QIV in the influenza seasons 2010/2011 to 2015/2016.

Measure	2010/2011	2011/2012	2012/2013	2013/2014	2014/2015	2015/2016	Total
Number of additional events avoided:							
Influenza cases	−	9,192	52,057	66,468	25,649	117,231	270,596
GP consultations	−	3,465	19,623	25,055	9,668	44,190	102,000
Workdays saved	−	4,758	26,945	34,404	13,276	60,679	140,062
Hospitalizations	−	113	639	816	315	1,439	3,323
Deaths	−	11	60	77	30	135	312
Third-party payer costs saved, Mex$:							
GP consultations	−	2,335,346	13,225,639	16,886,973	6,516,433	29,783,876	68,748,268
Hospitalizations	−	4,917,492	27,848,969	35,558,567	13,721,526	62,715,324	144,761,878
Total costs saved	−	7,252,838	41,074,608	52,445,539	20,237,959	92,499,201	213,510,146
Societal costs saved, Mex$:							
GP consultations	−	2,335,346	13,225,639	16,886,973	6,516,433	29,783,876	68,748,268
Productivity losses	−	1,585,455	8,978,819	11,464,480	4,423,974	20,220,123	46,672,850
Hospitalizations	−	4,917,492	27,848,969	35,558,567	13,721,526	62,715,324	144,761,878
Total costs saved	−	8,838,293	50,053,428	63,910,019	24,661,933	112,719,323	260,182,996

Abbreviations: GP, general practitioner; TIV, trivalent inactivated influenza vaccine; QIV, quadrivalent inactivated influenza vaccine; −, not applicable

The benefit of QIV was greater in seasons with a higher influenza attack rate (2013–2014 and 2015–2016) or with a higher proportion of influenza cases caused by a B-lineage mismatch with TIV (2012–2013) (Table 2 and Supplemental Table 1). For example, in 2015/2016, which had the highest influenza attack rate and a relatively high proportion of cases caused by the B lineage not included in TIV (Supplemental Table 1), the total third-party payer costs saved were Mex$92,499,201 – equivalent to 43.3% of all costs saved for the six included seasons (Table 2). There was no relative benefit of QIV over TIV in the 2010/2011 season, when all circulating B strains were of the same lineage as that included in TIV (i.e., TIV match = 100%).

**Figure 2.**
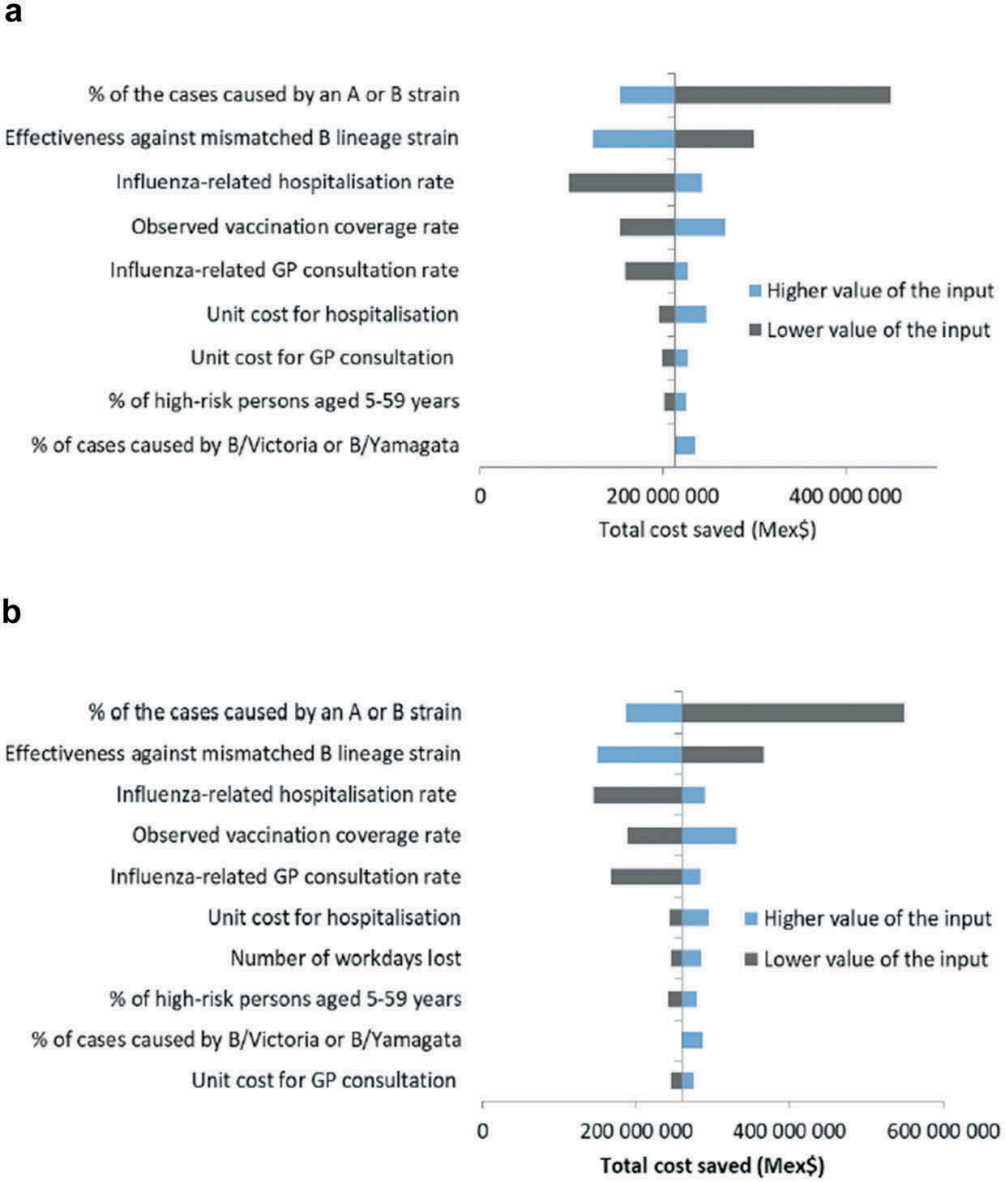
Deterministic sensitivity analyses of retrospective public health model. (A) Third-party payer perspective. (B) Societal perspective. Abbreviation: GP, general practitioner.

From the third-party payer perspective, the main drivers of the Reed-like model results were the distribution of cases attributable to A or B strains, the vaccine efficacy against the mismatched B strain (i.e., a higher level of cross-protection), the influenza-related hospitalization rate, the vaccination coverage, and the influenza-related GP consultation rate (Figure 2A). The relative benefit and associated cost savings of QIV increased with higher proportions of cases caused by B strains than by A strains, and with higher rates of influenza-related hospitalizations and GP consultations. By contrast, a greater efficacy of TIV against the mismatched strain in a given season decreased the relative benefit and costs saved with QIV. The unit cost for hospitalization, the proportions of cases caused by B/Yamagata and B/Victoria, the unit cost for GP consultations, and the proportion of high-risk individuals in the population also impacted the model results. The sensitivity analysis results were similar from the societal perspective, except that the number of lost workdays was an additional driver of the model (Figure 2B).

### Prospective impact of QIV in subsequent influenza seasons in mexico

Over the five subsequent influenza seasons from 2016/2017 to 2020/2021, assuming the epidemiological situation to be comparable to the previous seasons, a full replacement of TIV with QIV was estimated to prevent an additional 225,497 influenza cases, 85,000 influenza-related GP consultations, 116,718 days of influenza-related absenteeism, 2,769 influenza-related hospitalizations, and 260 influenza-related deaths (Supplemental Table 4). These avoided events were associated with cost savings of Mex$57,290,223 (US$2,911,237) from avoided GP consultations and Mex$120,634,899 (US$6,130,134) from avoided hospitalizations. Assuming that QIV and TIV are the same price per dose, the total costs saved by fully replacing TIV with QIV amounted to Mex$177,925,122 (US$9,041,370) for the healthcare system cumulated over 5 years. Under the societal perspective, including avoided losses in productivity due to absenteeism, the total cost saved for society over the 5-year period was equal to Mex$216,819,163 (US$11,017,794).

**Figure 3.**
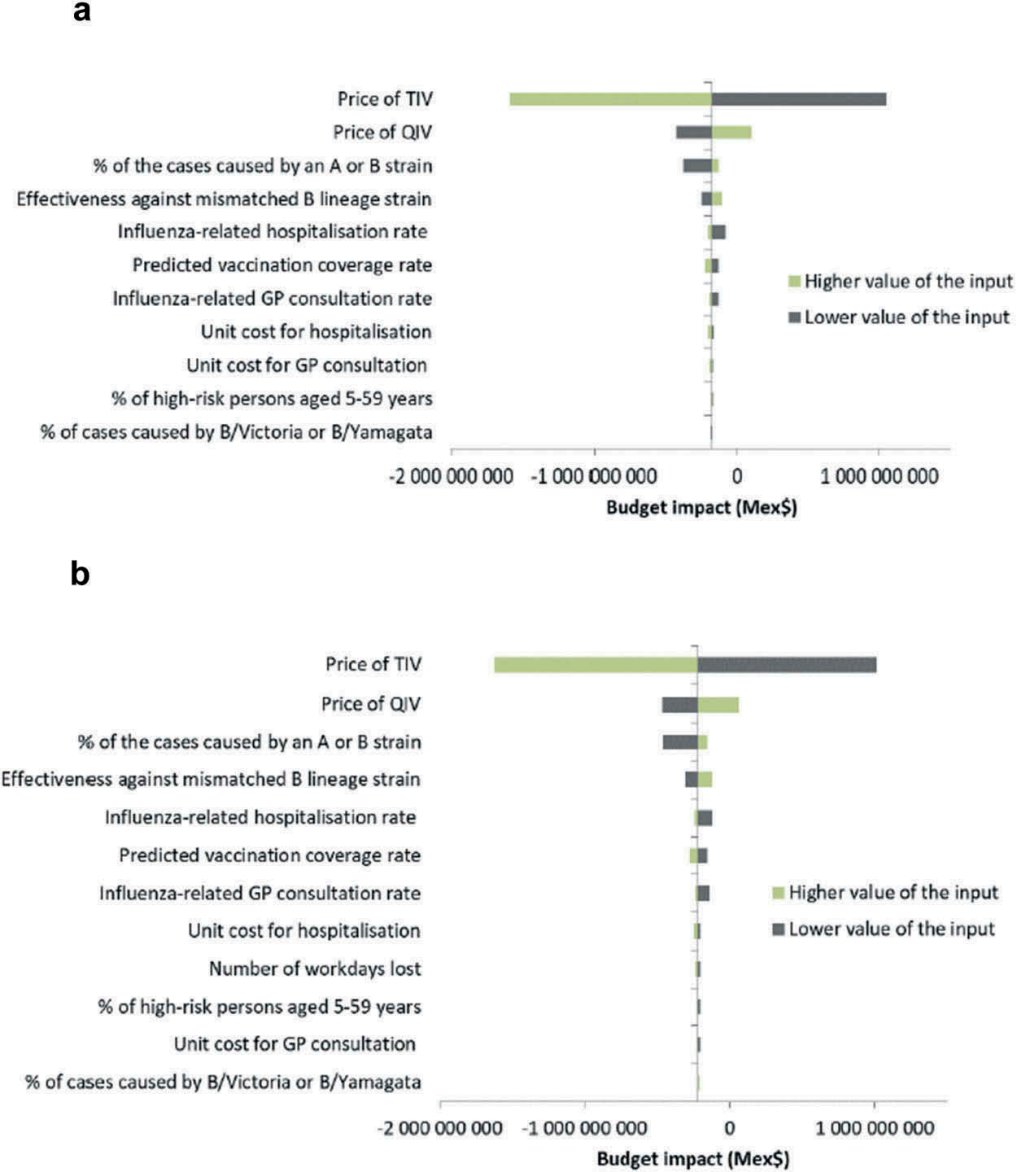
Deterministic sensitivity analyses of prospective budget impact model. (A) Third-party payer perspective. (B) Societal perspective. Abbreviations: GP, general practitioner; QIV, quadrivalent influenza vaccine; TIV, trivalent influenza vaccine.

From a third-party payer perspective, the main drivers of the budget impact model results were the vaccine prices and the proportion of cases due to A or B strains (Figure 3A). Variation in vaccine prices led to opposite outcomes: a price of TIV lower than QIV increased the budget impact of introducing QIV by increasing the vaccination costs. The relative benefits of QIV (and thus cost savings) increased with more influenza cases caused by B-strains. The total budget impact was also sensitive to TIV efficacy against the strain not included in the vaccine: a higher TIV efficacy against the mismatched B strain (i.e., a higher level of cross-protection) decreased the relative benefit and costs saved with QIV. Higher rates of influenza-related hospitalization or consultations also increased the relative benefit and costs saved with QIV. The sensitivity analysis results were similar from the societal perspective (Figure 3B).

## Discussion

Although influenza A viruses predominated in Mexico’s recent influenza seasons,^2,5^ considerable numbers of influenza B cases were also reported, particularly among children and adolescents.^5^ Currently, because TIVs are mainly used in Mexico, adequate protection against influenza B viruses relies on predicting the correct B lineage to include in each season’s influenza vaccine.^6^ Our public health impact and budget impact analyses showed that switching to QIV, which protects against both B lineages, over six previous influenza seasons in Mexico would have avoided considerable numbers of influenza cases, deaths, and lost workdays, and would have reduced the burden to the healthcare system. Together, this would have saved Mex$214 million (US$10.8 million) in third-party payer costs and Mex$260 million (US$13.2 million) in societal costs. Similarly, over five subsequent influenza seasons, the budget impact model showed that a full replacement of TIV with QIV in Mexico would save Mex$178 million (US$9 million) in third-party payer costs and Mex$217 million (US$11 million) in costs to society.

Most of the health benefits and costs saved with QIV were among young children and older adults. Similarly, young children and the elderly were estimated to benefit most from a switch to QIV in Brazil, Panama, and Columbia.^34^ The benefits of QIV for young children may be linked to the relatively high prevalence and considerable morbidity of influenza B infections in this age group.^5,7,35,36^

QIV resulted in the most avoided influenza-related events and cost savings in seasons that had higher incidence and greater mismatch between the TIV and the circulating B lineage (particularly 2015/2016, 2013/2014, and 2012/2013). QIV had less public health and economic benefits versus TIV in 2011/2012 and 2014/2015 – seasons that had lower attack rates and mismatch levels, and was of no benefit at all for the 2010/2011 season when all circulating B strains were of the same lineage as that included in TIV. The impact of QIV was similarly found to vary by season in three middle-income Latin American countries due to differences in intensity and vaccination coverage rates.^34^ Overall, the greatest benefits for the three countries were during the 2010 and 2012 seasons (corresponding to the Mexico 2009/2010 and 2011/2012 seasons), in which QIV was estimated to reduce influenza B cases by 8.2%−23.3% compared with TIV.^34^

Consistent with a systematic review of other health-economics studies,^9^ the relative benefit and cost savings of QIV were higher in seasons with more influenza B cases and with poor TIV efficacy against the mismatched B strain. In the retrospective model, the public health and economic benefits were also substantial when there were higher rates of influenza-related hospitalizations and GP consultations among the general population.

Our study was strengthened by including both retrospective and prospective models, that each covered multiple influenza seasons. The retrospective model also allowed us to investigate the public health and economic benefits in seasons with different attack rates and different levels of TIV B strain mismatch. However, both models had limitations. Because Mexico-specific data were unavailable for several model inputs, we had to use data obtained from other countries. For the 2011/2012 and 2014/2015 seasons, the distribution of cases caused by B/Yamagata and B/Victoria strains was based on data from the US, which may not have been representative. Indeed, the distribution of A/H1N1 and A/H3N2 cases was not equivalent between the US and Mexico during these two seasons,^37,38^ although we did not distinguish between A strains in our study. Similarly, no data were available from Mexico for influenza-related consultations, hospitalization rates, mortality rates, and lost workdays, meaning that estimates were derived from other countries and adjusted or assumed to be representative of the Mexican population.

A limitation in the budget impact model was that no price difference was assumed between the two vaccines. A recent systematic review on the health economic impacts of QIV identified seven studies in five high-income countries with published vaccine prices; the incremental vaccine price (in 2015 US $) of QIV over TIV was found to range from US $2 to US $5.^9^ A higher unit price for QIV compared to TIV in Mexico could impact the economic benefits, as suggested by the deterministic sensitivity analysis of our budget impact model. In addition, the population, population age structure, vaccination coverage, and outcome rates used for the prospective budget impact model were based on those for the retrospective model, assuming these factors would remain constant. The assumed static population could have underestimated the number of avoided events and costs saved, especially since recent trends show an increasing proportion of older adults among Mexico’s population.^13^ Similarly, large changes in vaccination coverage over future seasons could affect the budget impact, although we do not expect this to occur within the relatively short time frame used.

Due to limited available data, we were forced to make several other assumptions that might have impacted the model results. First, we were unable to include all of the potential high-risk groups among the population aged 5−59 years, which likely would have increased the estimated health benefits and cost savings with QIV. Second, because no specific vaccination coverage estimates were available for high-risk individuals aged 5−59 years, the coverage rate of the 60−64 year group was applied. However, this assumption was conservative since it was the lowest coverage estimate available for the target population. Third, our models used vaccine efficacy data from clinical trials, which can differ from the true vaccine effectiveness against individual influenza strains in a given season.^39^ Fourth, vaccination costs were assumed to be covered by the healthcare system, whereas employer-sponsored vaccination is also relatively common in Mexico. Finally, both models used a static rather than a dynamic approach, and so did not account for any herd effect from vaccination. For example, vaccination with QIV would have reduced overall influenza transmission of a mismatched B strain compared to TIV within Mexico, which could have increased the public health and economic benefits of switching to QIV.^35^

In conclusion, the results of the two models suggest that replacing TIV with QIV in Mexico’s vaccination program would prevent influenza cases and related GP consultations, absenteeism, hospitalizations, and deaths, thus leading to cost savings for both healthcare providers and society. These benefits could also encourage influenza vaccination in Mexico, which currently remains far below the WHO’s target of 75% for risk groups.^40^
