# Supplementary material for: Public health and economic impact of switching from a trivalent to a quadrivalent inactivated influenza vaccine in Mexico
Source: Hum Vaccin Immunother. 2019 Dec 18;16(4):827–35. doi: 10.1080/21645515.2019.1678997 (PMC7227722; doi:10.1080/21645515.2019.1678997)
Supplement: Supplemental Material [file khvi-16-04-1678997-s001.docx]

# Supplemental Materials for “Public health and economic impact of switching from a trivalent to a quadrivalent inactivated influenza vaccine in Mexico”

**Supplemental Table 1. Influenza attack rate, strain distribution, and outcome rates by age and season**

|  | **Influenza season** | | | | | |
| --- | --- | --- | --- | --- | --- | --- |
|  | **2010−2011** | **2011−2012** | **2012−2013** | **2013−2014** | **2014−2015** | **2015−2016** |
| Season severity coefficient^a^ | 0.88 | 0.98 | 0.52 | 1.46 | 0.55 | 1.61 |
| Attack rate, % |  |  |  |  |  |  |
| ≤4 years | 16.6 | 18.5 | 9.7 | 27.4 | 10.3 | 30.3 |
| 5−17 years | 14.5 | 16.2 | 8.5 | 24.0 | 9.0 | 26.6 |
| 18−49 years^b^ | 3.2 | 3.6 | 1.9 | 5.3 | 2.0 | 5.9 |
| 50−59 years^b^ | 3.2 | 3.6 | 1.9 | 5.3 | 2.0 | 5.9 |
| ≥60 years | 4.0 | 4.4 | 2.3 | 6.6 | 2.5 | 7.3 |
| Strain/lineage distribution, % |  |  |  |  |  |  |
| A/Any | 87.3 | 97.0 | 61.5 | 93.0 | 65.0 | 77.6 |
| B/Victoria | 12.7 | 1.5 | 15.5 | 7.0 | 7.2 | 11.2 |
| B/Yamagata | 0.0 | 1.4 | 23.0 | 0.0 | 27.8 | 11.2 |
| B lineage in TIV | Victoria | Victoria | Yamagata | Yamagata | Yamagata | Yamagata |
| Influenza-related GP consultations, N per 100,000 persons |  |  |  |  |  |  |
| ≤4 years | 5,233.6 | 5,833.8 | 3,060.3 | 8,623.7 | 3,240.6 | 9,561.4 |
| 5−17 years | 6,081.5 | 6,778.9 | 3,556.2 | 10,020.8 | 3,765.6 | 11,110.4 |
| 18−49 years^b^ | 1,322.2 | 1,473.8 | 773.2 | 2,178.7 | 818.7 | 2,415.5 |
| 50−59 years^b^ | 1,322.2 | 1,473.8 | 773.2 | 2,178.7 | 818.7 | 2,415.5 |
| ≥60 years | 1,813.9 | 2,021.9 | 1,060.7 | 2,988.8 | 1,123.1 | 3,313.8 |
| Influenza-related hospitalizations, N per 100,000 persons |  |  |  |  |  |  |
| ≤4 years | 234.1 | 261.0 | 136.9 | 385.7 | 145.0 | 427.7 |
| 5−17 years | 8.7 | 9.7 | 5.1 | 14.4 | 5.4 | 15.9 |
| 18−49 years | 13.5 | 15.0 | 7.9 | 22.2 | 8.4 | 24.6 |
| 50−59 years | 62.0 | 69.1 | 36.3 | 102.2 | 38.4 | 113.3 |
| ≥60 years | 138.7 | 154.7 | 81.1 | 228.6 | 85.9 | 253.5 |
| Influenza-related deaths, N per 100,000 persons |  |  |  |  |  |  |
| ≤4 years | 0.66 | 0.74 | 0.39 | 1.09 | 0.41 | 1.21 |
| 5−17 years | 0.15 | 0.16 | 0.09 | 0.24 | 0.09 | 0.27 |
| 18−49 years | 0.29 | 0.32 | 0.17 | 0.48 | 0.18 | 0.53 |
| 50−59 years | 4.30 | 4.80 | 2.52 | 7.09 | 2.67 | 7.86 |
| ≥60 years | 33.44 | 37.28 | 19.55 | 55.10 | 20.71 | 61.09 |

The average influenza attack rates for each age group were derived from the placebo arms of previous influenza vaccine trials.[^1-3^](#_ENREF_1) To obtain the rates for each season, the average attack rates were adjusted by the season severity coefficent^a^. The influenza-related general practitioner (GP) consultation rate, hospitalization rate, and death rate were derived from estimates from the US population[^4^](#_ENREF_4) because data for Mexico were not publically available. GP consultation rate estimates were adjusted by the ratio between the number of consultations per capita in Mexico and the US, obtained from 2011 statistics.[^5^](#_ENREF_5) Hospitalization and death rate estimates were not corrected for Mexico as it was assumed these are comparable to the US. Due to unavailability of specific data, influenza-related hospitalization and death rate estimates from the general population in Mexico were conservatively applied to high-risk individuals aged 5−59 years. Finally, each rate estimate was multiplied by the seasonal influenza attack rates to generate rates for each season. Abbreviations: QIV, quadrivalent influenza vaccine; TIV, trivalent influenza vaccine.

^a^ Season severity relative to the average severity over the modelled period. The value of the coefficient was calculated by dividing the number of cases reported at a given season by the average number of cases per season from the six seasons modelled. Source data were obtained from the FluNet database.[^6^](#_ENREF_6) If the coefficient value is greater than one, the season is more severe than average. If the coefficient value is smaller than one, the season is less severe than average.

^b^ Values are the same between the 18−49-year and 50−59-year groups because individual data for these age ranges were not available

**Supplemental Table 2. Incidence and prevalence input data by condition and age group**

| **Condition** | **Prevalence by age group (%)** | | | **Source** |
| --- | --- | --- | --- | --- |
|  | **5−17 years** | **18−49 years** | **50−59 years** |  |
| Respiratory conditions |  |  |  |  |
| COPD^a^ | 0.0 | 0.8 | 2.6 | [^7^](#_ENREF_7) |
| Asthma | 6.4 | 6.9 | 6.9 | [^8^](#_ENREF_8) |
| Cardio-vascular conditions |  |  |  |  |
| Stroke^b^ | 0.0 | 0.2 | 0.9 | [^9^](#_ENREF_9) |
| Angina | 0.0 | 0.5 | 2.0 | [^10^](#_ENREF_10) |
| Myocardial infarction^c^ | 0.0 | 0.4 | 1.7 | [^11^](#_ENREF_11) |
| Heart failure | 0.0 | 0.2 | 1.6 | [^12^](#_ENREF_12) |
| Metabolic conditions |  |  |  |  |
| Diabetes^d^ | 0.7 | 1.9 | 9.8 | [^13^](#_ENREF_13) |
| Obesity | 14.0 | 30.1 | 38.8 | [^14^](#_ENREF_14) |
| Immunity disorders & cancer |  |  |  |  |
| AIDS/HIV | 0.0 | 0.2 | 0.2 | [^15^](#_ENREF_15) |
| Cancer | 0.0 | 0.1 | 0.3 | [^16^](#_ENREF_16) |
| Chronic kidney disease | 0.0 | 1.7 | 4.3 | [^17^](#_ENREF_17) |
| Pregnancy | 0.0 | 3.4 | 0.0 | [^18^](#_ENREF_18) |
| Asplenia | 0.01 | 0.01 | 0.01 | [^19^](#_ENREF_19) |
| Sickle cell disease | 0.01 | 0.01 | 0.01 | [^20^](#_ENREF_20) |

Abbreviations: AIDS, acquired immunodeficiency syndrome; COPD, chronic obstructive pulmonary disease; HIV, human immunodeficiency virus

^a^ Corrected for double counting patients with asthma, assuming 40% of COPD patients also had asthma based on van de Molen et al.[^21^](#_ENREF_21)

^b^ Corrected for double counting patients with heart failure, assuming 16.5% of stroke survivors also have heart failure based on Balzi et al.[^22^](#_ENREF_22)

^c^ Corrected for double counting patients with angina, heart failure and stroke; assuming 26% of myocardial infarction survivors also have angina,[^23^](#_ENREF_23) 6.5% also have heart failure,[^23^](#_ENREF_23) and 8% have a history of stroke.[^22^](#_ENREF_22)

^d^ Corrected for double counting patients with obesity, assuming 41.9% of diabetes patients are also obese based on Nguyen et al.[^13^](#_ENREF_13)

**Supplemental Table 3. Retrospective outcomes prevented and costs saved by switching from TIV to QIV per age group**

|  | **Age group** | | | | |  |
| --- | --- | --- | --- | --- | --- | --- |
| **Measure** | **0−4 years** | **5−17 years** | **18−49 years** | **50−59 years** | **≥60 years** | **Total** |
| Number of additional events avoided, N (% total): |  |  |  |  |  |  |
| Influenza cases | 116,445 (43.0) | 55,923 (20.7) | 51,315 (19.0) | 13,010 (4.8) | 33,903 (12.5) | 270,596 (100) |
| GP consultations | 36,705 (36.0) | 23,385 (22.9) | 21,120 (20.7) | 5,355 (5.3) | 15,434 (15.1) | 102,000 (100) |
| Workdays saved^a^ | 30,832 (22.0) | 19,644 (14.0) | 57,025 (40.7) | 12,959 (9.3) | 19,602 (14.0) | 140,062 (100) |
| Hospitalizations | 1,642 (49.4) | 34 (1.0) | 216 (6.5) | 251 (7.6) | 1,181 (35.5) | 3,323 (100) |
| Deaths | 5 (1.6) | 1 (0.3) | 5 (1.6) | 17 (5.4) | 285 (91.3) | 312 (100) |
|  |  |  |  |  |  |  |
| Third-party payer costs saved, Mex$ (% total): |  |  |  |  |  |  |
| GP consultations | 24,739,298 (36.0) | 15,761,747 (22.9) | 14,235,216 (20.7) | 3,609,182 (5.2) | 10,402,825 (15.1) | 68,748,268 (100) |
| Hospitalizations | 51,227,750 (35.3) | 1,412,106 (1.0) | 9,070,240 (6.3) | 13,664,846 (9.4) | 69,386,936 (47.9) | 144,761,878 (100) |
| Total costs saved | 75,967,048 (35.6) | 17,173,853 (8.0) | 23,305,456 (10.9) | 17,274,028 (8.1) | 79,789,761 (37.4) | 213,510,146 (100) |
|  |  |  |  |  |  |  |
| Societal costs saved,  Mex$ (% total): |  |  |  |  |  |  |
| GP consultations | 24,739,298 (36.0) | 15,761,747 (22.9) | 14,235,216 (20.7) | 3,609,182 (5.2) | 10,402,825 (15.1) | 68,748,268 (100) |
| Productivity losses^a^ | 10,274,267 (22.0) | 6,545,877 (14.0) | 19,002,556 (40.7) | 4,318,255 (9.3) | 6,531,895 (14.0) | 46,672,850 (100) |
| Hospitalizations | 51,227,750 (35.3) | 1,412,106 (1.0) | 9,070,240 (6.3) | 13,664,846 (9.4) | 69,386,936 (47.9) | 144,761,878 (100) |
| Total costs saved | 86,241,315 (33.1) | 23,719,730 (9.1) | 42,308,012 (16.3) | 21,592,283 (8.3) | 86,321,656 (33.2) | 260,182,996 (100) |

Abbreviations: GP, general practitioner; TIV, trivalent inactivated influenza vaccine; QIV, quadrivalent inactivated influenza vaccine

^a^ Workdays saved and productivity losses in children reflected those incurred by their caregivers.

**Supplemental Table 4. Predicted impact of switching from TIV to QIV in the influenza seasons 2016−2017 to 2020−2021**

| **Measure** | **Average per season** | **Total (5 seasons)** |
| --- | --- | --- |
| Number of additional events avoided: |  |  |
| Influenza cases | 45,099 | 225,497 |
| GP consultations | 17,000 | 85,000 |
| Workdays saved | 23,344 | − |
| Hospitalizations | 554 | 2,769 |
| Deaths | 52 | 260 |
|  |  |  |
| Third-party payer costs saved, Mex$: |  |  |
| GP consultations | 11,458,045 | 57,290,223 |
| Hospitalizations | 24,126,980 | 120,634,899 |
| Total costs saved | 35,585,024 | 177,925,122 |
|  |  |  |
| Societal costs saved, Mex$: |  |  |
| GP consultations | 11,458,045 | 57,290,223 |
| Productivity losses | 7,778,808 | 38,894,042 |
| Hospitalizations | 24,126,980 | 120,634,899 |
| Total costs saved | 43,363,833 | 216,819,163 |
|  |  |  |

Abbreviations: GP, general practitioner; TIV, trivalent inactivated influenza vaccine; QIV, quadrivalent inactivated influenza vaccine; −, not applicable

**References**

1. Jefferson T, Di Pietrantonj C, Al-Ansary LA, Ferroni E, Thorning S, Thomas RE. Vaccines for preventing influenza in the elderly. Cochrane Database Syst Rev. 2010(2):CD004876.

2. Jefferson T, Di Pietrantonj C, Rivetti A, Bawazeer GA, Al-Ansary LA, Ferroni E. Vaccines for preventing influenza in healthy adults. Cochrane Database Syst Rev. 2010(7):CD001269.

3. Jefferson T, Rivetti A, Di Pietrantonj C, Demicheli V, Ferroni E. Vaccines for preventing influenza in healthy children. Cochrane Database Syst Rev. 2012(8):CD004879.

4. Molinari NA, Ortega-Sanchez IR, Messonnier ML, Thompson WW, Wortley PM, Weintraub E, Bridges CB. The annual impact of seasonal influenza in the US: measuring disease burden and costs. Vaccine. 2007;25(27):5086-96.

5. The Organisation for Economic Co-operation and Development. OECD data: doctor's consultations - total per capita, 2011. 2011 [accessed 16 Jan 2019]. https://data.oecd.org/healthcare/doctors-consultations.htm.

6. World Health Organization. FluNet. [accessed 22 Oct 2018]. <http://www.who.int/influenza/gisrs_laboratory/flunet/en/>.

7. Menezes AM, Perez-Padilla R, Jardim JR, Muino A, Lopez MV, Valdivia G, Montes de Oca M, Talamo C, Hallal PC, Victora CG. Chronic obstructive pulmonary disease in five Latin American cities (the PLATINO study): a prevalence study. Lancet. 2005;366(9500):1875-81.

8. Global Asthma Network. The Global Asthma Report 2014. Auckland, New Zealand: Global Asthma Network, 2014.

9. Kuri-Morales P, Emberson J, Alegre-Diaz J, Tapia-Conyer R, Collins R, Peto R, Whitlock G. The prevalence of chronic diseases and major disease risk factors at different ages among 150,000 men and women living in Mexico City: cross-sectional analyses of a prospective study. BMC Public Health. 2009;9:9.

10. Will JC, Yuan K, Ford E. National trends in the prevalence and medical history of angina: 1988 to 2012. Circ Cardiovasc Qual Outcomes. 2014;7(3):407-13.

11. Mitchell BD, Gonzalez Villalpando C, Arredondo Perez B, Garcia MS, Valdez R, Stern MP. Myocardial infarction and cardiovascular risk factors in Mexico City and San Antonio, Texas. Arterioscler Thromb Vasc Biol. 1995;15(6):721-5.

12. Bui AL, Horwich TB, Fonarow GC. Epidemiology and risk profile of heart failure. Nat Rev Cardiol. 2011;8(1):30-41.

13. Nguyen NT, Nguyen XM, Lane J, Wang P. Relationship between obesity and diabetes in a US adult population: findings from the National Health and Nutrition Examination Survey, 1999-2006. Obes Surg. 2011;21(3):351-5.

14. Barquera S, Hernández-Barrera L, Pedroza-Tobías A, Rivera-Dommarco JA. Prevalencia de obesidad en adultos mexicanos, ENSANUT 2012. Salud Publica Mex. 2013;55(2):151-60.

15. CentroNacional para la Prevención y elControl del VIH/SIDA. La epidemia del VIH y Sida en México. 2015.

16. International Agency for Research on Cancer. GLOBOCAN 2012: Estimated Cancer Incidence, Mortality and Prevalence Worldwide in 2012 [accessed 9 Jan 2019]. <http://publications.iarc.fr/Databases/Iarc-Cancerbases/GLOBOCAN-2012-Estimated-Cancer-Incidence-Mortality-And-Prevalence-Worldwide-In-2012-V1.0-2012>.

17. Coresh J, Selvin E, Stevens LA, Manzi J, Kusek JW, Eggers P, Van Lente F, Levey AS. Prevalence of chronic kidney disease in the United States. JAMA. 2007;298(17):2038-47.

18. República Mexicana Consejo Nacional de Población. Nacimientos por grupos de edad de la madre, 2010-2050. 2017 [accessed 9 Jan 2019]. https://[www.gob.mx/conapo](http://www.gob.mx/conapo).

19. Osorio-Díaz JO. Síndrome de Heterotaxia Visceral asociado a una cardiopatía compleja cianógena de lujo pulmonar aumentado y bloqueo atrioventricular completo: reporte de caso. Revista de Medicina e Investigación. 2013;1(1):31-3.

20. Hassell KL. Population estimates of sickle cell disease in the U.S. Am J Prev Med. 2010;38(4 Suppl):S512-21.

21. van der Molen T, Miravitlles M, Kocks JW. COPD management: role of symptom assessment in routine clinical practice. Int J Chron Obstruct Pulmon Dis. 2013;8:461-71.

22. Balzi D, Barchielli A, Buiatti E, Franceschini C, Lavecchia R, Monami M, Santoro GM, Carrabba N, Margheri M, Olivotto I, et al. Effect of comorbidity on coronary reperfusion strategy and long-term mortality after acute myocardial infarction. Am Heart J. 2006;151(5):1094-100.

23. Longmore RB, Spertus JA, Alexander KP, Gosch K, Reid KJ, Masoudi FA, Krumholz HM, Rich MW. Angina frequency after myocardial infarction and quality of life in older versus younger adults: the Prospective Registry Evaluating Myocardial Infarction: Event and Recovery study. Am Heart J. 2011;161(3):631-8.
